# Respiration organizes gamma synchrony in the prefronto-thalamic network

**DOI:** 10.1038/s41598-023-35516-7

**Published:** 2023-05-26

**Authors:** Diellor Basha, Sylvain Chauvette, Maxim Sheroziya, Igor Timofeev

**Affiliations:** 1 Département de Psychiatrie Et de Neurosciences, Université Laval, Québec, QC G1V 0A6 Canada; 2 CERVO Centre de Recherche, Université Laval, 2301 Av. D’Estimauville, Québec, QC G1E 1T2 Canada

**Keywords:** Non-REM sleep, REM sleep, Slow-wave sleep, Wakefulness, Cellular neuroscience, Neural circuits

## Abstract

Multiple cognitive operations are associated with the emergence of gamma oscillations in the medial prefrontal cortex (mPFC) although little is known about the mechanisms that control this rhythm. Using local field potential recordings from cats, we show that periodic bursts of gamma recur with 1 Hz regularity in the wake mPFC and are locked to the exhalation phase of the respiratory cycle. Respiration organizes long-range coherence in the gamma band between the mPFC and the nucleus reuniens the thalamus (Reu), linking the prefrontal cortex and the hippocampus. In vivo intracellular recordings of the mouse thalamus reveal that respiration timing is propagated by synaptic activity in Reu and likely underlies the emergence of gamma bursts in the prefrontal cortex. Our findings highlight breathing as an important substrate for long-range neuronal synchronization across the prefrontal circuit, a key network for cognitive operations.

## Introduction

The prefrontal cortex (PFC) is an anatomical hub that integrates inputs from a diverse set of cortical and subcortical structures^1^. Much of PFC output is reciprocated^1^ with the notable exception of hippocampal afferents^2,3^ for which no direct prefrontal feedback projections have been found. The nucleus reuniens (Reu), a midline thalamic structure, provides a crucial link between the PFC and the hippocampus, forming a functional network that mediates multiple cognitive operations^4–6^. Precise, inter-regional synchronization in this network is essential for memory processes and is largely facilitated by oscillations that emerge during different states of consciousness^7–9^.

The gamma rhythm (30–80 Hz), which is prominent in the aroused brain^10^, plays an important role in the normal physiology^11^ and pathophysiology^12^ of the prefrontal network. It has been consistently linked to higher-level functions such as consciousness^13^, attention^14^ and memory^15,16^, which denotes its fundamental role in prefrontal cortical functions^11,17^. The ability of gamma to organize cortical activity is closely related to its 25 ms cycle which provides an optimal window for the co-activation of multiple neurons within successive cycles, facilitating the formation of local neuronal assemblies^18,19^.

There has been a recent accumulation of evidence showing that respiration is a potent modulator of cortical and hippocampal activity^20–24^. In the rodent^21,25,26^ and cat cerebral cortex^27^, respiration modulates gamma amplitude and elicits a respiration-related cortical rhythm (RR), which tends to be more prominent in frontal regions^20,21,28^. How respiration organizes gamma synchrony in the prefrontal network is unknown. It has been shown that Reu mediates long-range gamma synchrony between the PFC and hippocampus^29^ and appears to be necessary for the emergence of slow potentials in the PFC^30^ that are likely respiration-related^31,32^. We thus hypothesized that respiratory signals relayed through Reu organize gamma synchrony in the prefrontal network. Given the dramatic changes in neuronal excitability and the emergence of different brain rhythms across states of vigilance, we further hypothesized that respiration-gamma coupling is modulated by brain state.

## Results

To study the dynamics of gamma synchrony in the prefrontal network, we recorded local field potentials (LFPs) from deep layers of the cat mPFC, midline thalamus and the ventral hippocampus in head-fixed configuration (Supplementary Fig.S1a and S1b). The animals cycled through periods of quiet wakefulness, non-rapid eye movement (NREM) and rapid eye movement (REM) sleep over sessions of 2–4 h (Supplementary Fig. S1c and S1d). Additionally, we performed in vivo intracellular recordings of the midline thalamus and of the prefrontal cortex in anesthetized mice (Fig. 4 and Supplementary Fig. S4a).

### Selective modulation of gamma synchrony in prefronto-thalamic networks by respiration

**Figure 1:**
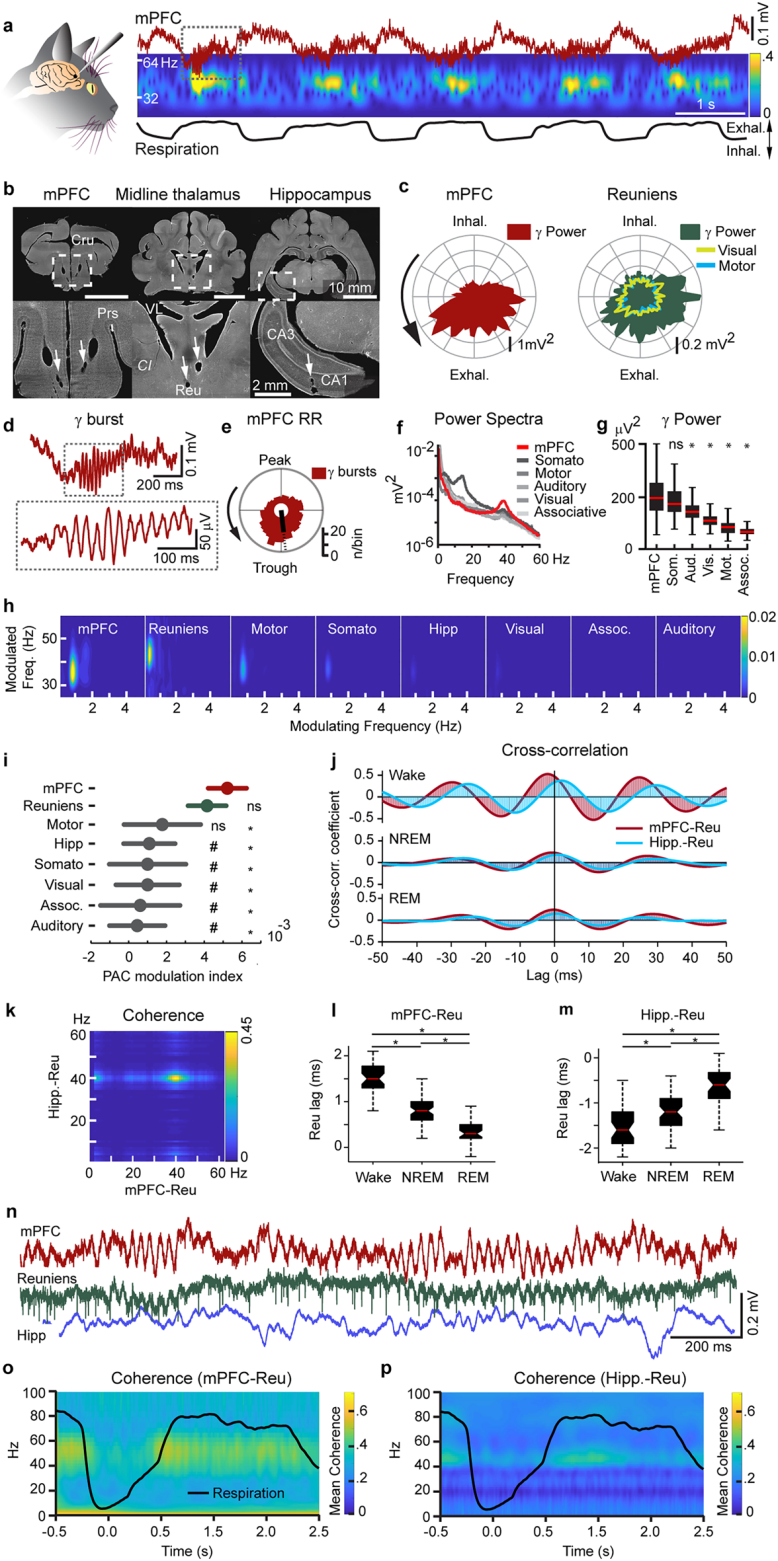
Respiration modulates gamma synchrony in the prefronto-thalamic network. (**a**) mPFC local field potential and its wavelet spectrogram showing transient gamma oscillations nested in the respiration-related potential (RR). Dotted box denotes the burst shown in this figure (**d**). (**b**) DAPI-labelled, coronal sections showing electrode tracks in the deep layers of the mPFC, the midline thalamus and the CA1 region of the hippocampus. *Cru–sulcus cruciatus, Prs–sulcus praesylvius, CI–capsula interna, Reu–nucleus reuniens*. (**c**) Prefrontal (left) and Reu (right) gamma power versus phase of respiration. (**d**) An expanded view of a single gamma burst from this figure (**a**). (**e**) Polar plot showing the phase preference of gamma bursts to the trough of the respiration-related potential (RR) for all detected gamma bursts. The black line is the mean resultant vector (p_Rayleigh_ < 0.001). (**f**) Power spectra from multiple cortical areas during wake episodes. (**g**) Gamma power was significantly higher in the mPFC than in other cortical sites (one-way Tukey-Kramer post-hoc ANOVA,* p* < 0.0001). (**h**) Example comodulograms, showing the strength of phase-amplitude coupling in various recorded sites during wake. (**i**) Population data of modulation indices calculated from all recordings. Gamma-RR PAC was higher in the mPFC than in all other recorded areas (except RE, post-hoc ANOVA,* p* <0.001). Gamma-RR PAC in Reu was significantly higher than in the hippocampus, somatosensory, visual, association and auditory cortex but not motor or mPFC (post-hoc ANOVA, *p* < 0.001). (**j**) Example cross-correlograms computed from bandpassed (30-80 Hz) signals of the mPFC, Reu and hippocampus in wake, NREM and REM states. Cross-correlogram were computed for mPFC-Reu pairs s (red) and hippocampus-Reu pairs (blue). (**k**) Signal coherence between the mPFC and Reu and between Reu and the hippocampus was highest in gamma and RR range. (**l**) Reu lagged the mPFC by a significantly longer latency in wake epochs than in NREM and REM. (**m**) Reu led the hippocampus by a significantly longer latency in wake epochs than in NREM and REM. (**n**) An example recording segment showing relatively high synchrony in the mPFC-thalamo-hippocampal network during periods of gamma activity. (**o**) The mean wavelet coherence between the mPFC and Reu, centered to the onset of exhalation (time = 0 s) showing the modulation of prefronto-thalamic gamma coherence by respiration phase. (**p**) The mean wavelet coherence between Reu and the hippocampus, centered to the onset of exhalation (time = 0 s) showing the modulation of thalamo-hippocampal gamma coherence by respiration phase.

Our initial analysis of the mPFC signal revealed rhythmic variations in gamma power resulting from brief gamma bursts (382 ± 90 ms, 332 ± 46 µV^2^, Fig. 1a and d) which recured every 1–2 s and were phase-locked to the envelope of a 1-Hz, prefrontal rhythm (Fig. 1a and e, Rayleigh Uniformity test, p_Rayleigh_ < 0.001). A few studies have indicated that oscillatory activity in prefrontal networks is modulated by respiration^21,25,33^. We thus asked whether the 1-Hz modulation of gamma power observed in our recordings is related to respiration and whether this is unique to prefrontal-thalamic networks. We found that the 1-Hz rhythm was coherent with respiration, with gamma-expressing troughs occurring during exhalation (Fig. 1a and d). We refer to this 1-Hz potential henceforth as the respiratory-related rhythm (RR), reflecting similar terminology used by Biskamp, et al.^21^ and Mofleh and Kocsis^24^.

Phase-amplitude coupling between RR phase and gamma was significantly more pronounced in the mPFC and Reu than in the hippocampus, somatosensory, suprasylvian (associative), ectosylvian (auditory), marginal (visual) but not motor cortex (Fig. 1c,h,i, Tukey–Kramer post-hoc ANOVA, hereafter post-hoc, *p * < 0.0001). Close inspection of mPFC, Reu and hippocampal signals revealed that wake activity is characterized by periods of strong coherence between the three structures in the gamma range (Fig. 1k,n). Prefronto-thalamic (Fig. 1o) and thalamo-hippocampal (Fig. 1p) coherence in the gamma range was significantly higher at the exhalation phase of respiration, dipping to a minimum during inhalation (t-test,* p* < 0.0001).

We considered the possibility that gamma oscillations are volume-conducted from other brain regions to Reu. To this end, we computed cross-correlations from wake, NREM and REM recordings and measured the mPFC-Reu and hippocampus-Reu time lag in each state. In wake, Reu signals lagged the mPFC by a significantly longer delay than in NREM and REM and Reu signals led the hippocampus by a significantly longer delay during wake than during NREM and REM (Fig. 1j–m), suggesting that gamma activities recorded in Reu are physiological phenomenon, but not volume conducted.

### Respiration-gamma coupling is modulated by states of consciousness

**Figure 2:**
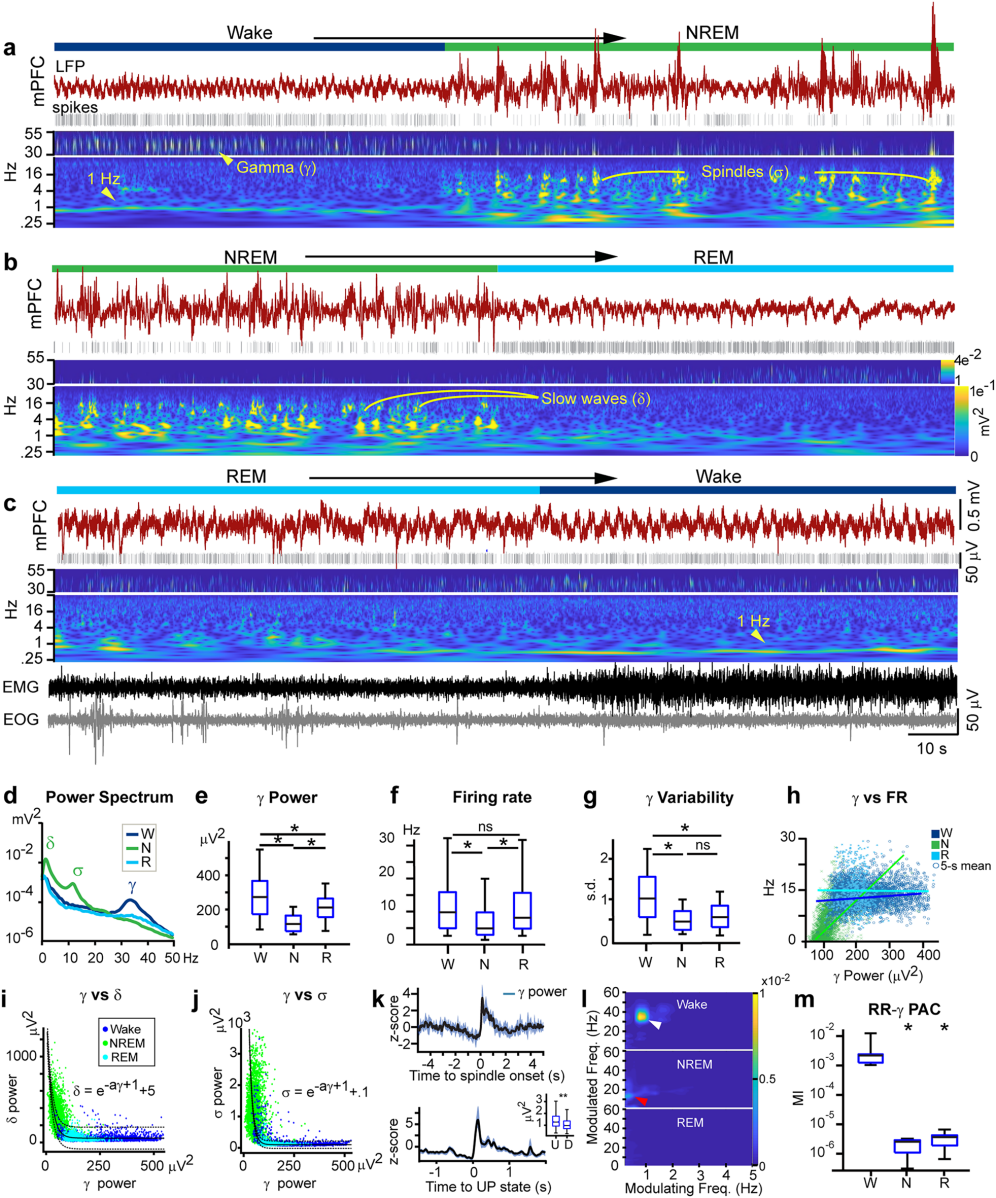
Respiration-gamma coupling is selective to wakefulness. (**a**) LFP recording of the mPFC during a wake-to-NREM transition (top) and its wavelet transform (bottom) showing the attenuation of gamma bursts during the transition. (**b**) NREM-to-REM transition. Sigma and delta power attenuated as the mPFC transitioned to REM. (**c**) REM-to-wake transition. The 1-Hz, RR oscillation in the mPFC signal emerged in wake although overall gamma power remained comparatively similar to REM levels. (**d**) The Fourier transform of the mPFC signal from one recording session, separated according to states of vigilance. Wakefulness was characterized by a peak in the 30–40 Hz gamma range, NREM by high delta and sigma power and REM by a low, broad peak in gamma. (**e**) mPFC gamma power was highest in wakefulness (one-way Tukey-Kramer post-hoc ANOVA, *p* < 0.0001). (**f**) mPFC firing rates were highest in wake and REM and lowest in NREM (ANOVA, *p* <0.001). (**g**) Variability of gamma power in 5-second windows across states of vigilance, measured as standard deviations. (**h**) Gamma power versus firing rate across states of vigilance, plotted as measurements in 5-second windows. (**i**) Delta power decayed exponentially with the emergence of gamma in REM and wake (nonlinear least-square regression, r = 0.92). Dots are 5-second means of gamma or delta-band power in the mPFC signal. (**j**) Sigma power decayed exponentially with the emergence of gamma in REM and wake (nonlinear least-squares regression, r = 0.97). (**k**) Normalized gamma power measurements, centered to the onset of spindles, showing increase gamma power during spindles (top). Normalized gamma power measurements, centered to the start of UP states (down). Data are mean ± s.d. Inset: gamma power was significantly higher during UP states compared to down (ANOVA, *p* <0.001). (**l**) Phase-amplitude coupling of the mPFC in wake, NREM and REM states. White arrow indicates RR-gamma coupling and red arrow indicates slow-oscillation-spindle coupling. (**m**) PAC modulation indices in different states, calculated for gamma amplitude (30–60 Hz) and phase of the respiration-related oscillation (0.2–2 Hz). RR-gamma modulation was higher in wakefulness than in NREM and REM (post-hoc ANOVA, *p* <0.001).

Next, we examined the evolution of gamma oscillations over states of consciousness and its interplay with other electrophysiological rhythms. The wavelet-transform of the mPFC signal revealed that RR-gamma coupling diminished rapidly as the brain transitioned into NREM, a state that was predominated by delta (1–4 Hz) and sigma (10–16 Hz) frequencies (Fig. 2a). Transitioning from NREM to REM, overall gamma power increased steadily although we observed neither rhythmic variability of gamma power nor the RR measured during wake (Fig. 2b and c). Overall, delta (r = 0.92) and sigma (r = 0.97) power diminished at an exponential rate with the emergence gamma oscillations in REM and wake (Fig. 2i and j).

After segmenting the recordings into periods of wake, NREM and REM (see State detection and Supplementary Fig. S2a), we first calculated the spectral content of the mPFC signal using the fast Fourier transform. As expected, dominant frequencies during NREM were in the delta and sigma range (Fig. 2d), reflecting the underlying slow oscillation and spindle events, respectively (Fig. 2a and b). Wake, and to a lesser extent, REM, were characterized by a peak in 30–50 Hz gamma (Fig. 2d). We found that gamma power was significantly higher and more variable during wake than during REM and NREM (Fig. 2e and g, post-hoc,* p* < 0.0001) with the lowest gamma power occurring during NREM (p < 0.001).

Using template matching and principal component analysis, we extracted single-unit data from mPFC and midline thalamic recordings (Supplementary Fig. S3b). mPFC firing rates were highest in wake and REM and lowest in NREM (Fig. 2f., post-hoc ANOVA,* p* < 0.001), mirroring the gamma profile of the signal (Fig. 2e). We therefore considered the possibility that signal power in the gamma band emerged from underlying multiunit activity^34–36^. To address this, we examined the relationship between mPFC gamma power and mPFC spiking and found that during NREM, mPFC firing rates were linearly predicted by gamma power (Fig. 2h, r(2998) = 0.59, *p* < 0.0001). However, mPFC firing rates were not correlated with mPFC gamma power (r(3159) = 0.06, *p* = 0.11) neither during wake nor during REM (r(668) = 0.02, *p*  = 0.54), suggesting the presence of a field gamma rhythm, independent of multiunit activity.

Spectral power analysis showed that gamma oscillations were significantly diminished in NREM sleep (Fig. 2e). However, within this bracket of low gamma power in NREM, we detected weak but notable increases in the gamma band during DOWN-UP transitions and spindles (Fig. 2a,b,k). Measurements of power in the period around spindles and slow waves revealed a significant increase in the gamma band at the onset of spindles (Fig. 2k) and at the onset of the UP state (Fig. 2k (inset), *p* < 0.001). Phase-amplitude coupling (PAC) analysis showed that RR-gamma coupling is selective to wake states and does not occur in NREM or REM (Fig. 2l and m, post-hoc ANOVA, *p* < 0.001). PAC in NREM was characterized by delta phase modulation of sigma amplitude, reflecting the coupling of spindles to the slow oscillation. We found no discernable modulation of gamma rhythms in REM by other frequencies.

### Respiration modulates midline thalamic firing

**Figure 3:**
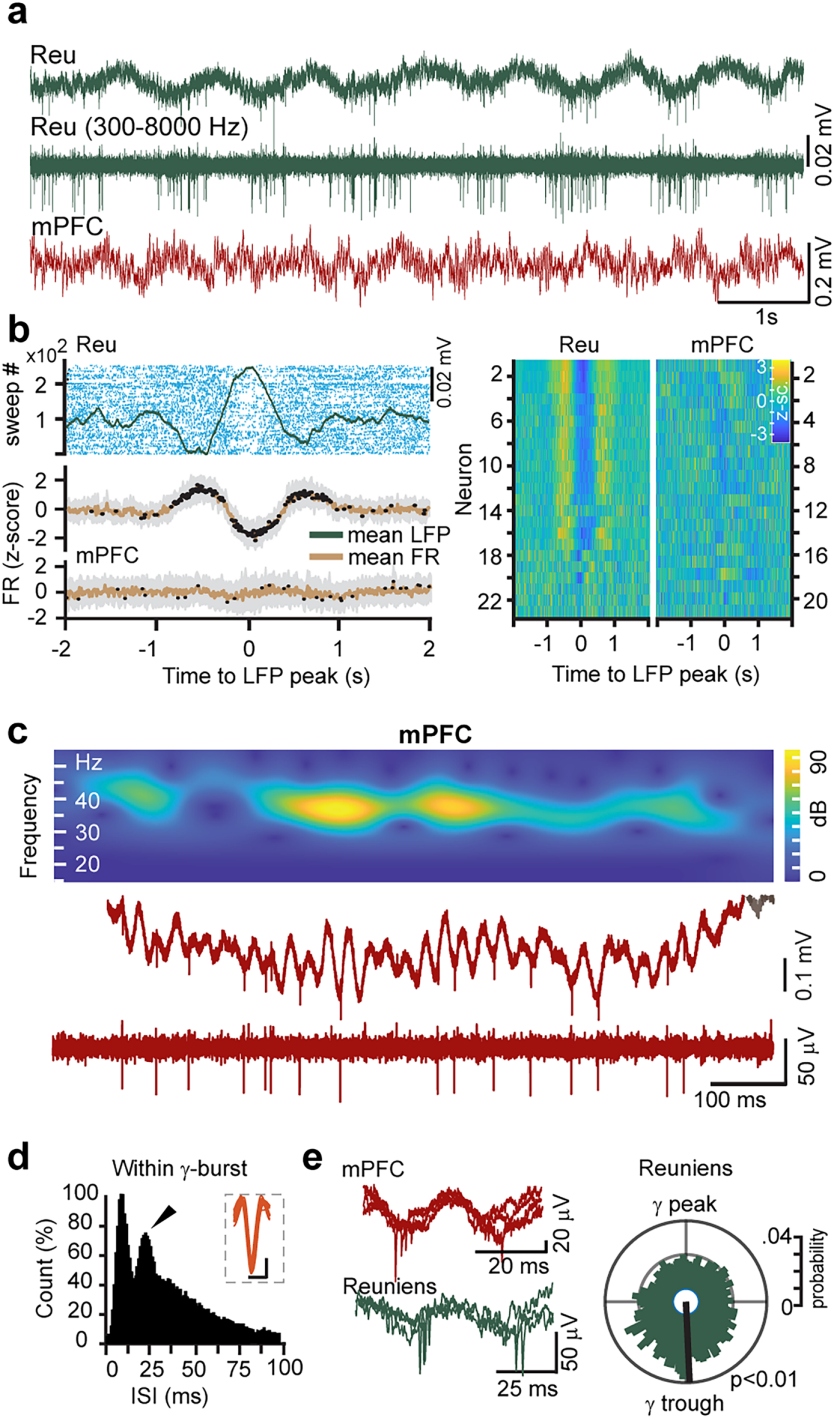
Respiration modulates firing in the nucleus reuniens. (**a**) Example recording segment showing the modulation of Reu multi-unit activity by the phase of respiration-related potential. (**b**) The respiration-related potential modulates the firing rate of Reu but not mPFC single-units. From top to bottom: Peri-event firing rate dynamics of a Reu single-unit centered to the peak of the LFP respiration-related oscillation. Dots are single-unit discharges organized from bottom to top as sweeps around the onset of the LFP peak. The shaded area is the firing rate mean ± s.d. of 23 Reu and 21 mPFC single-units, normalized to the mean firing rate of each unit. Black dots indicate bins that were significantly different (t-test, *p* < 0.01) in comparison to equivalent bins obtained from shuffled peri-event spike histograms. Right: firing rate dynamics of each Reu and mPFC single-units, referenced to the respective LFP peak and normalized to the mean firing rate of each unit. (**c**) Expanded view of a single gamma burst recorded from the cat mPFC, showing the LFP gamma oscillation and the associated single-unit activity, phase-locked to gamma cycles. Red trace is the mPFC signal and its bandpass (300-8000 Hz, below). The spectrogram (top) shows the wavelet transform of the trace. (**d**) The interspike interval distribution of a prefrontal single-unit within gamma bursts. Note the ~25 ms interspike intervals indicative of gamma periodicity. Inset shows several traces of the detected spike. Horizontal bar is 1 ms, vertical bar is 20 µV. (**e**) Example traces of mPFC and reuniens spike/LFP recordings showing the phase preference of prefrontal and reuniens single units for the trough of gamma cycles (left). Polar plot shows the firing probability of a reuniens single-unit on the phase of the gamma cycle (right).

Next, we looked at the modulation of prefrontal and thalamic firing by gamma and respiration phase. Reu single units were significantly modulated by the RR (3a and 3b), showing decreased firing probability at the peak of the thalamic LFP oscillation (Fig. 3b). We found no RR modulation of mPFC firing rates (Fig. 3b) although, within prefrontal gamma bursts, the distribution of mPFC interspike intervals showed a prominent peak at the ~ 25 ms mark, indicative of gamma periodicity (Fig. 3c and d) and Reu single-units showed phase preference for the trough of the gamma cycle (Fig. 3e, p_Rayleigh_ < 0.01).

### Respiration drives synaptic activity in the nucleus reuniens

**Figure 4:**
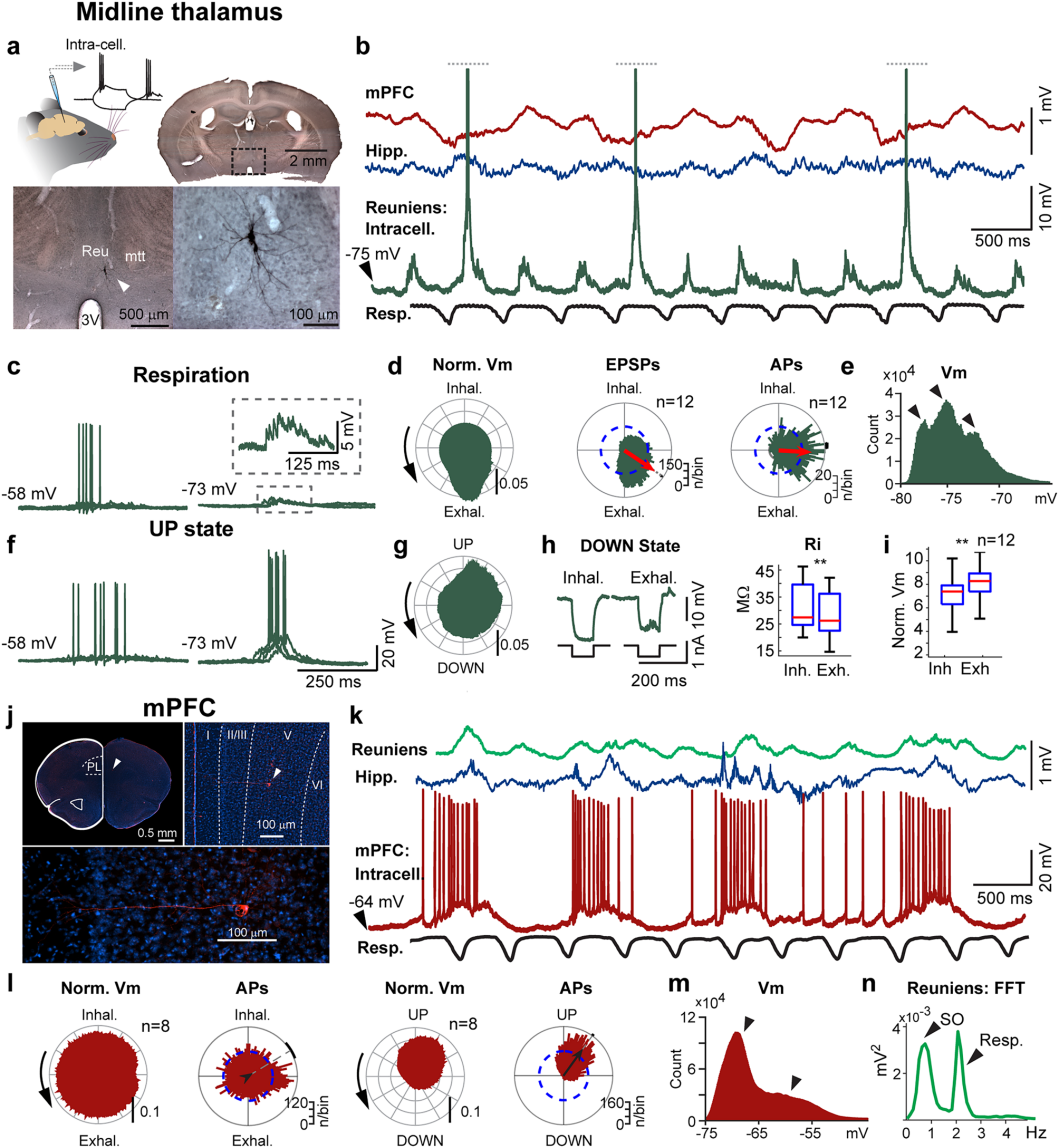
Respiration and the slow oscillation comodulate synaptic activity in the nucleus reuniens. (**a**) Coronal section of the ventral midline thalamus showing a reuniens neuron labelled with neurobiotin, revealed immunohistochemically by DAB horseradish peroxidase. *Reu–nucleus reuniens; mtt–mamillothalamic tract; 3V–ventral third ventricle. *(**b**) In vivo intracellular recording of a reuniens cell labelled in A. (**c**) Reuniens activity during exhalation in the DOWN state of the slow oscillation at basal and hyperpolarized levels (−0.6 nA and −1.5 nA current injection). (**d**) Reuniens intracellular events show preference to exhalation phase. Membrane depolarization, EPSPs and action potential discharges were non-uniformly distributed around respiration phase (Rayleigh test, *p* < 0.001 for EPSP and *p* <0.001 for action potentials, n = 12). (**e**) Tri-modal distribution of the membrane potential, indicating hyperpolarizing and depolarizing events typical of the slow oscillation (left and right arrows) and respiration-related EPSPs (middle arrow). (**f**) Reuniens activity during the cortical UP state. Under hyperpolarizing current injection, UP states induced low-threshold spikes that were not observed during respiration cycles. (**g**) The Reu membrane potentials was modulated by the slow oscillation. (**h**) Hyperpolarizing pulses given in DOWN states, during inhalation and exhalation. The membrane voltage response to the same current injection was smaller during exhalation. Input resistance (Ri) was higher during inhalation than exhalation. (**i**) The membrane potential was significantly higher during the exhalation phase than during inhalation (t-test, *p* < 0.001). (**j**) A layer V pyramidal neuron of the mPFC (prelimbic cortex) labelled with neurobiotin, revealed immunohistochemically by streptavidin-Texas Red^TM^ conjugate. *PL – prelimbic cortex; I, II/III, V, VI - cortical laminae*. (**k**) In vivo intracellular recording of a prefrontal cortical cell labelled in J. (**l**) mPFC intracellular activity is locked to UP states but not to respiration From left to right: uniform membrane depolarization and action potential discharge around respiratory phase (*p*_Rayleigh_= 0.494, n = 12). Non-uniform membrane depolarization and action potential discharge around slow oscillation phase (*p*_Rayleigh_<0.0001, n = 12). (**m**) Bi-modal distribution of the membrane potential of the mPFC cell labelled in J, indicating hyperpolarizing and depolarizing events typical of the slow oscillation. (**n**) Spectral content of the reuniens LFP signal showing comodulation of the local field by the slow oscillation and the respiratory cycle.

Considering the modulation of Reu single-unit activity by the RR, we asked whether respiration impacts membrane potential dynamics of Reu cells and how this relates to gamma. To this end, we performed in vivo intracellular recordings of Reu in mice under ketamine-xylazine (Fig. 4a and b), an anesthetic that boosts cortical gamma and slow oscillations^37^. We found that the intracellular activity of Reu was modulated both by the respiratory cycle (Fig. 4c–e) and the slow oscillation (Fig. 4f and g), reflecting concomitant gamma dynamics in the mPFC signal (Supplementary Fig. S4). In agreement with a previous study^38^, prefrontal gamma oscillations were modulated by the slow oscillation, increasing with UP states and decreasing with DOWN states (Supplementary Fig. S4). However, compared to the somatosensory EEG signal, the mPFC also exhibited transient increases in gamma amplitude outside of UP states, which were phase locked to the respiratory cycle (Supplementary Fig. S4a and S4b). Reu neurons were depolarized and often fired action potentials during exhalation (Fig. 4b,d and Supplementary Fig. S4a and S4b), preceding the gamma maximum in the LFP of the mPFC (Supplementary Fig. S4a and S4b). In the hippocampus, the main structure influenced by respiratory cycles was the dentate gyrus, which showed phase-locked firing to respiration and strong modulation of gamma power with exhalation (Supplementary Fig. S4d and S4g). The respiration-related modulation of LFP dynamics in the dentate gyrus was strongly dependent on input from the ipsilateral olfactory bulb and level of anesthesia (Supplementary Fig. S5). Ablation of the olfactory bulb ipsilateral to the recorded dentate gyrus resulted in a strong attenuation of respiratory potentials (Supplementary Fig. S5a and S5b). Similarly, the respiration rhythm in the dentate gyrus was diminished in light anesthesia (Supplementary Fig. S5c–S5f.). In the CA1, the main determinants of firing rate were internal events such as UP states (Supplementary Fig. S4i) and sharp-wave ripples (Supplementary Fig. S4f. and S4j) but not respiration (Supplementary Fig. S4h).

We considered the possibility that neuronal activities correlated with respiration were a result of movement artefacts. To address this issue, we measured the input resistance of Reu neurons during inhalation and exhalation. We found decreased input resistance during exhalation (Fig. 4h), suggesting that exhalation is associated with synaptic drive in Reu. Next, we detected spontaneous excitatory synaptic events (Supplementary Fig. S3a). These synaptic events peaked at the transition from exhalation to inhalation and were followed by action potentials (Fig. 4d). We conclude that Reu neurons receive excitatory synaptic drive coupled with respiration. This synaptic drive triggers spiking of Reu neurons which contribute to the control of respiration-related gamma activity in mPFC.

## Discussion

We studied the relationship between respiration and gamma oscillations in the mPFC, midline thalamus, hippocampus and other cortical areas in head-restrained cats cycling through wake, NREM and REM states and in mice under ketamine-xylazine anesthesia. Our data reveal (1) the existence of a distinct respiration-related potential in the mPFC and the midline thalamus (2) recurring prefronto-thalamic gamma synchrony, phase-locked to exhalation in wakefulness and deep anesthesia, but not NREM and REM sleep (3) the modulation of Reu synaptic activity by the respiratory cycle and (4) a selective dependence of respiration-gamma coupling on the wake state. Taken together, our data demonstrate that respiration synchronizes activity via gamma oscillatory mechanisms in the prefrontal network, a circuit responsible for multiple cognitive functions.

The origin of the respiratory drive to Reu and the mPFC is uncertain. In the nasal epithelium, rhythmic airflow is known to activate mechanoreceptors, synchronizing neuronal activity that drives downstream oscillations in olfactory structures such as the olfactory bulb^39^ and the piriform cortex^40^. The piriform cortex sends projections directly to the PFC^41^ and indirectly to the hippocampus via the entorhinal cortex^42,43^ but, notably, there are no direct piriform projections to Reu^44^. Our observation of large, sharp-rising EPSPs generated during respiratory cycles indicate that the respiratory input to Reu is likely due to “driver” excitatory synapses that form on proximal dendrites of the thalamus^45–47^. For higher-order nuclei, such inputs originate primarily in layer V pyramidal neurons of the cortex. However, sharp-rising EPSPs in Reu often occurred during prefrontal silence in DOWN states, excluding any role for prefrontal firing in the generation of ‘driving' EPSPs in this thalamic nucleus. Within the hippocampal formation, the strongest olfactory modulation is recorded in the dentate gyrus with weakening responses in the neighbouring CA1^48^. Since the primary hippocampal inputs to Reu arise in CA1/subiculum with no projections from the dentate gyrus^44^, it is unlikely that the hippocampus is the primary source of respiratory signals to Reu. Out of multiple direct inputs to Reu^49^, the most probable contributor to the respiratory rhythm is the entorhinal cortex which receives direct piriform input^43^ and projects to Reu^44^.

The activity of inputs from respiratory or olfactory structures does not explain why the respiratory rhythm was present in wake and deep anesthesia, but absent during NREM and REM, when breathing remained regular. Respiration and cortical potentials are spectrally coherent during wake but not coherent during NREM and REM^27^. Although respiratory frequency in cats depends on behavioural state^50^, we did not detect respiration-related oscillations in the cortical LFP during NREM and REM. This implies the involvement of brainstem neuromodulators that control sleep–wake states. The cholinergic system, which is active during both wake and REM sleep^51^ is unlikely to be involved in RR-gamma coupling which was most prominent in wake but not REM. The dopaminergic system, which projects to the frontal cortex, but not other cortical areas^52^ is a likely mediator especially since dopaminergic neurons tend to burst fire selectively during wakefulness to facilitate dopamine release^53^. Additionally, ventral tegmental dopaminergic neurons send projections to Reu^49^. Serotoninergic neurons of the raphe nucleus project to Reu^44^, are involved in the control of respiration^54^ and the vast majority of these neurons fire during wakefulness^55^. However, the serotoninergic system has brain wide projections, but we observed respiratory modulation of the gamma rhythm primarily in the frontal cortex and a lesser extent in somatosensory cortex (Fig. 1). It is therefore likely that the modulation of the prefrontal gamma rhythm is mediated by an interplay of respiratory, olfactory, dopaminergic and serotoninergic structures.

The synaptic source of gamma oscillations recorded from the thalamus is ambiguous. In laminar structures such as the cortex and the hippocampus, the recurrent axial geometry of pyramidal cells gives rise to a consistent laminar profile of current dipoles that are generated by transmembrane currents. The algebraic superposition of these currents across a large number of neurons gives rise to a coherent field that can oscillate in the case of rhythmic synaptic activity^56^. The thalamus does not have a laminar organization, but it has some degree of morphological and functional assimetry^57,58^. It is unclear whether transmembrane currents coalesce in the thalamus to give rise to a coherent field oscillation. Therefore, we considered the possibility that gamma oscillations in our recordings of the cat thalamus are volume conducted from neighbouring regions or from laminar structures such as the cortex. Considering that volume conduction is a passive electrical phenomenon, we hypothesized that the relative time lag between cortical and thalamic signals should remain constant and should not be affected by behavioural states. Analysis of time-lags showed that the relative synchronicity between mPFC-Reu and hippocampus-Reu signals changed between wake, NREM and REM states, which lead us to conclude that thalamic gamma is dependent on physiological mechanisms.

In cats, the closest cortical structure to Reu is 12 mm dorsal and is separated from it by the 3rd and lateral ventricles as well as the large callosal and capsular fibre bundles^59^. Reu is surrounded by other thalamic nuclei in its dorsal and lateral extent and is bordered ventrally by the ventral 3rd ventricle^59^. In addition, the cat thalamus has abundant GABAergic interneurons that produce IPSPs in thalamocortical cells^60^. Since the local generation of local oscillations almost always depends on excitatory-inhibitory interplay^18^, it is possible that the cat thalamus and that of other large mammals can sustain fast oscillations. Accordingly, gamma oscillations can be observed in the membrane potential of thalamocortical neurons in cat^61^. In humans, fast field oscillations with phase-locked spiking in beta and gamma range have been recorded in non-layered deep structures including the thalamus^62–64^, subthalamic nucleus^65,66^ and the pallidum^63,67^. While it is true that the biological source of the thalamic field in cats and humans is unclear, volume conduction is unlikely considering the large volume of anisotropic tissue between the cortex and subcortical structures, interspersed by grey matter, fluid-filled ventricles and fiber bundles. In addition, gamma bursts in mPFC were not synchronized with gamma range activities in anterior suprasylvian gyrus, located at distances shorter than 10 mm. In contrast, the rodent thalamus has a negligible number of local inhibitory interneurons^68^ and may not be able to generate fast oscillations. Indeed, analysis of the current-source density of field oscillations in the mouse reuniens results in a flat signal^69^, suggesting the absence of local field oscillations. The exception is the lateral geniculate, which has 20–30% inhibitory interneurons^68^ and here, thalamocortical gamma synchrony has been reported in mice for both LFP and spikes^70^. Apart from the presence of local inhibitory interneurons, the reuniens of cats is largely homologous to the limbic connectivity of the reuniens in mice. Retrograde labelling of cell bodies was found in the reuniens following tracer injections in the hippocampus^26^ while anterograde tracer injections in the reuniens resulted in terminal labeling in CA1 region and subiculum^21^. Similar bidirectional connectivity exists between the reuniens and the mPFC^71–73^, with greater reuniens connectivity in the infralimbic (area 25) area of the mPFC compared to the prelimbic (area 32). Since the field is generated by transmembrane currents that arise primarily due to postsynaptic potentials, thalamic gamma oscillations in the cat Reu are thus most likely due to cortical or hippocampal afference^74,75^.

The ability of neurons to time their spike discharges with millisecond precision is thought to be important for the coding of information^76,77^. Accordingly, the emergence of gamma oscillations within a structure provides transient opportunities for precise, coordinated firing among partner neurons^18^. We found, that within the RR-nested gamma bursts, spike timing was restricted to the gamma cycle (Fig. 3), leading to patterned firing in the gamma frequency range (~ 25 ms ISI), both for mPFC and Reu cells. Of notable interest was the decreased spiking probability at the ~ 17 ms mark which is suggestive of either GABA_A_-mediated inhibition or refractory periods, as predicted by computational models^78,79^. Although the RR does not alter cortical firing rates as it does in Reu, its nested gamma bursts provide periods of temporal order that permit the mPFC to overcome the natural low correlation of spike discharges prevalent in the waking cortex^80^. Such network synchrony likely integrates the diverse information streams that reach the mPFC from the thalamus, hippocampus and subcortical regions. Given that gamma activity between cortical areas and their corresponding thalamic nuclei is highly correlated^81^, we propose that these RR-nested gamma bursts facilitate the cognitive functions of the mPFC by forming the basis for long-range coordination in the prefrontal network. Among the many cognitive operations mediated by prefrontal gamma oscillations, attention can be singled out as an inherently wake-dependent phenomenon that is closely linked to gamma oscillations and the PFC^82–85^.

We considered the possibility that respiratory modulation during ketamine-xylazine anesthesia may be an artefact arising from respiration-associated muscle activity or electrode displacement. To address this caveat, we performed unilateral lesions of the olfactory bulb during recordings of the hippocampal formation (Supplementary Fig. S5). Olfactory bulbectomies produced a significant reduction in the respiratory potential observed in hippocampal LFP during anesthesia, quantified by cross-correlation of the respiratory signal and the hippocampal LFP. The data reiterates findings from other studies^21,22^ which showed that respiratory potentials in the brain are abolished by olfactory bulbectomy. In addition, the emergence of respiratory potentials in the hippocampus and the cortex was strongly dependent on the level of anesthesia, arising during deep anesthesia and diminishing in light anesthesia, suggesting a physiological mechanism independent of electrode stability. Similarly, there was a marked attenuation of respiratory potentials in cat NREM sleep despite the stable breathing rate and maintained muscle tone. Lastly, the phase of respiration determined the firing rate and the input resistance of Reu which suggests a synaptic drive associated with respiration. We conclude that Reu neurons receive an excitatory synaptic drive coupled with respiration which contribute to the control of respiration-related gamma activity in the mPFC.

## Methods

### Experimental model and subject details

Experiments were carried out in accordance with the guidelines of the Canadian Council on Animal Care and were approved by the Committee for Animal Care of Université Laval (Comité de protection des animaux de l’Université Laval CPAUL) and are consistent with ARRIVE guidelines.

In five adult cats (4 females, aged 1–1.5 years), the LFP was recorded from the hippocampus, midline thalamus, and various cortical areas including mPFC, somatosensory, motor, auditory, associative and visual cortex; see Surgeries for stereotaxic coordinates).

In vivo intracellular recordings of the thalamus were obtained from 12 adult C57BL6 mice (male, aged 3–6 months), anesthetized with ketamine-xylazine, alongside LFP recordings of the mPFC and the ventral CA1 region of the hippocampus. In a second group of mice (n = 11, male, aged 3–6 months), in vivo intracellular recordings of the mPFC were obtained together with LFP recordings of the midline thalamus and the ventral hippocampus.

### Surgeries

#### Cats

Surgical procedures for the implantation of electrodes in cat were carried out according to methods described previously in Chauvette et al.^37^,Timofeev, et al.^86^. Briefly, cats were pre-anesthetized with an intramuscular injection of ketamine (3 mg/kg), Buprenorphine (0.02 mg/kg), and Dexmedetomidine (5 µg/kg). The incision site was shaved, and the cats were intubated for gaseous anesthesia. Lidocaine (0.5%) and bupivacaine (0.25%) was injected at the site of the incision and in all pressure points where the head contacted the stereotaxic frame. Four stainless-steel electrodes were implanted under stereotaxic guidance in the mPFC (*from the interaural line: AP 23 to 24 mm, ML 1 mm, DV 5 mm, θ* = *10°*) according to the Reinoso-Suarez stereotaxic atlas of the cat brain^59^. The four mPFC electrodes were arranged as two sets of stereotrodes, each set sheathed within a 21-gauge cannula, oriented anteroposteriorly (between AP = 23 and AP = 24 mm). Additionally, single stainless-steel electrodes were implanted in deep cortical layers (1.2 mm from cortical surface) in the ipsi- or contralateral hemisphere in the marginal gyrus [visual cortex (areas 17 and 18)], suprasylvian gyrus [associative cortex (areas 5, 7, and 21)], ectosylvian gyrus [auditory cortex (areas 22 and 50)], postcruciate gyrus [somatosensory cortex (area 3)], precruciate gyrus [motor cortex (areas 4 and 6)] (Supplementary Fig. S1a). Tetrodes, constructed out of four twisted microwires (platinum-iridium, 12.5 µm, gold-plated to ~ 200 kΩ), were implanted into the midline thalamus (*from the interaural line: AP 11.5, ML 0.5, DV 1.5, θ* = *10°*) and into the CA1 region of the hippocampus (*from the interaural line: AP 7, ML 10.5, DV −3.5, θ* = *10°*).

For electro-oculography (EOG), a silver electrode was implanted onto the orbital bone and for electromyography (EMG), two electrodes were implanted into the neck muscle. All recordings were referenced to a silver electrode fixed to the skull above the cerebellum. All electrodes, wires and connectors were fixed in acrylic dental cement. To allow for subsequent head-restrained recordings, a head-fixation holders were also attached and encased into the dental cement. Throughout the surgery, the body temperature was maintained at 37 °C using a water-circulating thermo-regulated blanket. Heartbeat, oxygen saturation and blood pressure were continuously monitored using a pulse oximeter (Rad-8; MatVet), and the level of anesthesia was adjusted to maintain a heartbeat at 130–150 per minute. Lactate Ringer’s solution was given intravenously (5 ml/ kg/h, i.v.) during the surgery. After the surgery, cats received meloxicam (0.05 mg/kg) once a day for 3 days.

#### Mice

Mice were anesthetized with ketamine and xylazine (100 and 10 mg/kg, i.p.), placed on a heated platform (37 °C) and fixed onto a stereotaxic frame. Deep anesthesia was maintained with supplemental doses of ketamine-xylazine, administrated every 30–40 min. The skull was exposed, and small craniotomies were made above the mPFC (*from bregma: AP* + *1.8, ML* + *0.5 mm*), midline thalamus (*AP −1.0, ML 0.5 mm*), hippocampus (*AP −3.3; ML −3.3 mm*), contralateral somatosensory cortex (*AP −1.5, ML 2.5* mm) and above the cerebellum (midline of interparietal bone, *AP −6 to −8, ML 0*). The dura was cut using a 23-gauge needle and mineral oil (Sigma-Aldrich Inc.) was applied over the exposed cortex to prevent drying. To minimize pulsations arising from intracranial pressure variations, the fourth ventricle was opened by cannulation of the cisterna magna. One stainless steel screw (1 mm diameter) was fixed into the craniotomy over the somatosensory cortex (EEG) and one over the cerebellum (reference electrode). Monopolar, tungsten microelectrodes (10–12 MΩ, FHC, Bowdoin, US) were implanted into the infralimbic/pre-limbic area of the mPFC (*from bregma: AP* + *1.8, ML* + *0.5, DV 2.0, θ* = *5º*) or in the reuniens nucleus (*AP −1, ML* + *0.5, DV 4.2, θ* = *5º*) and into the intermediate/ventral hippocampus (*AP −3.25, ML* + *3.5, 2.0* < *DV* < *3.0, θ* = *5º*). In 5 mice, bulbectomies were performed during LFP recordings of the dentate gyrus (AP -2.2, ML + 1.0, DV 2.0) by unilateral aspiration of the olfactory bulb accessed through a craniotomy above the olfactory bulb (*AP* + *5, ML* + *0.5, DV 1.0).*

### Electrophysiological recordings

#### Natural sleep and wake in cats

A recovery time of 1 week was maintained prior to the first recording session. Cats were trained over 2–3 days to remain in head-restrained position for 2–4 h and cycle through periods of quiet wakefulness, NREM, and REM sleep. Recordings were obtained over two weeks following the post-operative recovery period. All recordings were conducted within a Faraday chamber using AM 3000 amplifiers (A-M Systems), bandpassed 0.1 Hz to 10 kHz with a 1 k gain. Respiration was recorded using a thermocouple (TAC80B-K, Omega) placed near the animal’s nostrils. Exhalation and inhalation phases were determined according to temperature changes produced near the nostril. Exhalation was recorded by the thermocouple as a temperature-dependent voltage increase and inhalation as a decrease. All signals were sampled at 20 kHz and digitized with PowerLab (ADInstruments—Data Acquisition Systems for Life Science, RRID:SCR_001620).

#### In vivo intracellular recordings in mice

Intracellular recordings were obtained in vivo in mice using glass micropipettes pulled on a vertical puller (Narishige PP-830, Tokyo, Japan) from borosilicate capillary tubes (WPI; P-97, Sutter Instrument) and filled with 2–3% neurobiotin (Sigma) in 2 M potassium acetate. Two regions were targeted: the nucleus reuniens (*from bregma: AP −1.5, ML* + *0.5, 3.9* < *DV* < *4.4, θ* = *5º*) or the medial prefrontal cortex (*from bregma: AP* + *1.8, ML* + *0.5, DV 2.0, θ* = *5º*). The micropipette was advanced dorsoventrally until a cell was entered, confirmed in situ by electrophysiological observations (i.e., low-threshold spikes characteristic of the thalamus; see Supplementary Fig. S3). For recording stability, craniotomies were sealed with 4% agar in saline. A high-impedance amplifier (Neurodata IR-283 amplifiers; Cygnus Technology) with active bridge circuitry was used to record the membrane potential (sampled at 20 kHz) and to inject current into neurons.

LFPs of the mPFC, reuniens and the hippocampus were obtained using monopolar tungsten microelectrodes (11–12 MΩ). The CA1 microelectrode was advanced into the hippocampus in 50 µm increments until sharp wave ripples (SWRs) were observed, confirming entry in the pyramidal layer of the CA1 region. EEG recordings of the somatosensory cortex were obtained from the stainless-steel screw. Respiration was recorded using a piezoelectric sensor strip placed on the animal’s thorax such that chest expansion with inhalation produced rising values and exhalation produced falling values. LFP and EEG signals were amplified 1000 times, sampled at 10 kHz for LFP and 1 kHz for EEG, bandpassed 0.1 Hz to 10 kHz using AM 3000 amplifiers (A-M Systems) and digitized using PowerLab (ADI Instruments).

Following recording sessions (5–45 min), cells were iontophoretically labelled with Neurobiotin by injecting positive current pulses (200 ms on–off duty cycle, 0.5–1.5 nA, 10 min) through the recording pipette. Approximately 30 min after labeling, mice were perfused intracardially with 4 °C saline (0.9%) followed by 4% paraformaldehyde (PFA) in 0.1 M phosphate buffer (PB). Brains were extracted, stored in PFA for a minimum of 24 h post-fixation and sliced coronally into 70–80 µm-thick sections.

### Histology

To verify stereotaxic targeting in cats, visualization of electrode tracks in histological sections was performed at the conclusion of recordings. The animals were deeply anesthetized with a large dose (0.5 mL) of ketamine-xylazine (i.m., 15 and 2 mg/kg) and euthanized by intracardial perfusion of cold 0.9% saline followed by 4% paraformaldehyde (PFA). Brains were extracted and placed in several sucrose-PFA solutions of increasing sucrose concentration (10, 20 and 30% sucrose in PFA). The brain was then sliced in a freezing microtome and sections were processed in 3 batches with consecutive sections going to Nissl-staining, DAPI (300 nM) and antifreeze solution. Nissl-stained sections were coverslipped in Permount (Fisher Scientific Inc.), DAPI sections were coverslipped in Dako fluorescence mounting medium (Sigma-Aldrich Inc.) and the remaining sections were cryoprotected in antifreeze and stored for future histological analysis. The coverslipped sections were visualized in brightfield (Nissl) or fluorescence (DAPI) microscopy and sections with electrode tracks were photographed in 10 and 20 × magnification.

Neurobiotin-labelled cells were revealed either by DAB-peroxidase reaction or by streptavidin-Texas Red™ conjugate. For DAB revelation, free-floating sections were incubated in ABC kit (1:200; Vector Laboratories) in Tris-buffered saline (TBS) for 2 h, rinsed and then incubated in TBS containing 0.05% DAB (Sigma), 0.005% CoCl_2_, and 0.00125% H_2_O_2_ for 5–10 min. For streptavidin-Texas Red™ revelation, sections were incubated in 1:250 dilution of streptavidin-Texas Red™ (Invitrogen, S872) in PB and then rinsed. Sections were visualized and photographed in brightfield microscopy for DAB-staining and in laser-scanning, fluorescence microscopy for streptavidin-Texas Red™.

### Analysis

Signals were analyzed using built-in and custom routines in Spike2 (Cambridge Electronic Design, Ltd.) and custom-written code in MATLAB (Mathworks Inc.), available at https://github.com/DiellorBasha/MATLAB_for_Electrophysiology.

### State detection

Detection of wake, NREM and REM sleep was based on automated analysis of the mPFC, and EMG signals followed by manual REM scoring using mPFC, EMG and EOG data. For automated detection, the Spike2 script RatSleepAuto, based on rodent recordings^87^, was adapted to cat mPFC recordings. Briefly, the mPFC signal was downsampled to 100 Hz and bandpassed in the delta range (1–4 Hz). The mPFC, EMG and the delta-filtered mPFC signals were then squared and the mean of each signal was calculated in 5 s bins. Each 5-s bin was classified as NREM if the mPFC and delta signals exceed the mean + 1 standard deviation (s.d.) of the signal and if the EMG fell below mean—1 s.d. All bins other than NREM were initially classified as wake. In the subsequent manual correction, “wake” bins with EMG below mean–1 s.d. and EOG above mean + 1 s.d. were classified as REM (Supplementary Fig. S2). In the final stage, the 5-s bins were combined into groups of 4 to create 20 s epochs of wake, REM or NREM according to the majority of 5-s bins that comprised the 20 s epoch. Epochs without a majority of either NREM, REM or wake were labelled as “Transition”.

### Spectral analysis

To obtain the spectral content of the LFP, the fast Fourier transform (FFT) was calculated within each state of vigilance for all channels (Fig. 1f.) using built-in spectral analysis functions in Spike2. The resultant FFTs were accumulated and averaged to obtain the mean power spectrum for each state of vigilance. Power in the gamma band (30–55 Hz) was calculated for each channel in 5-s epochs and the average value of gamma power within each state of vigilance was obtained (Fig. 2e and Supplementary Fig. S1e). To analyze the variability of gamma power in the signal, the standard deviation of gamma power within a 5-s epoch was calculated for each state of vigilance (Fig. 2g). To determine the evolution of spectral features in mPFC signal over time, magnitude scalograms of the signal (Fig. 1a and 2a–c) were obtained using the Morse continuous wavelet transform (*cwt* in MATLAB). The mean values of gamma were calculated in 5 s windows and plotted against the 5-s means of delta (Fig. 2i) and sigma power (Fig. 2j). Exponential decay functions were fitted to these data using nonlinear least-squares regression in the MATLAB Curve Fitting toolbox (robust, trust-region-reflective method).

### Gamma burst detection

Recurring bursts of gamma (Fig. 1a and d) during wakefulness were detected as peaks in the mPFC gamma band. The mPFC signal was downsampled to 1000 Hz, bandpassed (30–55 Hz) and the root mean square (RMS, τ = 0.025) of the bandpassed signal was calculated. Peaks of amplitude 0.025 mV or higher in the RMS signal were detected and timestamped. Considering that gamma bursts recurred in 0.5–1.5 s intervals (Fig. 1a,h, Fig. 2l), the slow envelope (< 5 Hz) of the mPFC channel was calculated to determine the phase of the LFP at which gamma bursts occurred. The signal was downsampled to 100 Hz and lowpassed < 5 Hz and troughs in the lowpassed LFP were detected. The trough-to-trough intervals were used as the 0-to-2π period over which the phase the LFP was estimated. The time of the gamma peak was converted to phase values within the 0-to-2π period and the resultant phase values were binned (Fig. 1e).

### Spindle and slow wave detection

Spindles were analyzed by first detecting bursts of LFP power in the spindle frequency band (10–15 Hz) using mean + 0.5 s.d. as the detection threshold (SleepSpindle04 script in Spike2). The detected spindles were then revised in 3 steps: episodes shorter than 300 ms were excluded; spindles that occurred within 300 ms of each other were combined into a single event and spindles longer than 3 s were excluded.

Slow waves were detected as zero-crossings around high-amplitude peaks in the mPFC signal within previously labelled NREM periods. First, the mPFC signal was downsampled by a factor of 200 (final sampling frequency–100 Hz) and the signal was detrended (DC remove, τ = 1) and lowpassed between 1 and 8 Hz. Peak amplitudes larger than 150 µV with a maximum width of 200 ms were marked only within NREM epochs. Next, the nearest zero crossings within 500 ms of the peak were detected as onsets and offsets of the slow wave. Rising zero crossing within 250 ms before the peak were marked as slow wave onsets (signifying the beginning of the DOWN state) and falling zero crossing within 250 ms after the peak were marked as slow wave offsets (signifying the beginning of the UP state). Gamma power in the UP state (200 ms window) was then measured and compared to gamma power during the DOWN state (200 ms window).

### Spike detection and analysis

Single-units were detected from the mPFC signal using the built-in, template-matching algorithm in Spike2. Briefly, fast signals (~ 1–3 ms) that exceeded a threshold were first detected as putative spikes. Thresholds were manually determined according to the signal-to-noise ratio of spikes in each recording. Next, a temporary spike template was formed according to the shape of the putative spike and a “template width” was estimated (twice the mean difference between the template and the spike that created it). Each new spike was then compared against the template and added to it if the spike’s sample points fell within the template width. The template was modified with the addition of each new spike up to a maximum of eight spikes after which the template was checked against previous templates. If a match was found, the temporary template was merged with the existing template. Otherwise, a new confirmed template was generated. After template matching, spikes were assigned into separate classes according to clusters formed by their principal components. Well-separated clusters derived from principal component analysis (PCA) were considered as single units (Supplementary Fig. S3). All other units were excluded from the analysis.

Peri-event time histograms (PETH) were computed in Spike2 to determine firing rate changes around gamma bursts or peak RR. Peaks in gamma power or LFP during wakefulness (gamma bursts) were considered as zero times for the PETH. Around each detected event, a 3-s sweep was passed through the spike channel and the spikes over all sweeps were accumulated into 1 ms bins to form a time histogram of spike counts. Finally, to determine spike rates (spikes/second or Hz), the number of spikes in each bin was first divided by the number of sweeps and then divided by the bin width. The interspike-interval (ISI) histogram was calculated using standard methods of counting the ISIs and accumulating the result to form a histogram. Spike autocorrelations were calculated using a similar approach to PETH where 100 ms sweeps of the spike channel were triggered by spike itself (zero-time reference).

### Intracellular data analysis

Action potentials were detected as peaks in the membrane potential (Vm) signal 10 s.d. above the mean. Similar to previous study^88^, to detect EPSPs, the Vm was lowpassed (< 1000 Hz) and differentiated to obtain dV/dt values. Peaks above mean + 1 to 3 s.d. of the differentiated signal were detected as EPSPs (Supplementary Fig. S3).

### Phase, coherence and cross-correlation analysis

To estimate the phase of the respiration, the respiration signal was lowpassed (< 5 Hz) and angle of the Hilbert transform was taken (Supplementary Fig. S2b). Phase values were then binned into 360 bins between 0 and 360 degrees (or - π to π). Similar methods were used to determine the phase of the respiratory-related potential from LFP signals (lowpassed < 5 Hz), the slow oscillation phase in anesthesia recordings (lowpassed < 2 Hz) and the gamma phase (bandpassed 30–60 Hz).

To determine the amplitude of the gamma oscillation, LFP signals were bandpassed (30–55 Hz) and the magnitude of the Hilbert transform was taken. Membrane potential values from intracellular recordings were detrended, normalized to 100% and the magnitude of the Hilbert transform was taken. The magnitude data were binned according to respiration or slow oscillation phase bins (see above) and the mean value per bin was calculated. The mean gamma or Vm magnitude per bin was plotted against respiration phase or against the slow oscillation phase to obtain a phase modulation profile (Supplementary Fig. S2b, right). To calculate the phase of spike or EPSP occurrence, timestamps obtained from spike/EPSP detection were used to evaluate the phase of the slow oscillation or gamma cycles at that time point.

For phase amplitude coupling, comodulograms and modulation indices were obtained from continuous wake recordings using methods developed by Tort et al.^89^ and the corresponding MATLAB toolbox (https://github.com/tortlab/phase-amplitude-coupling). Briefly, continuous signals were filtered in the RR band (0.2–2.5 Hz) and in the gamma band (30–60 Hz) and the modulation indices were computed as a measure of PAC magnitude, taking RR as the “phase-modulating” frequency and gamma as the “amplitude-modulated” frequency (Fig. 1i). To visualize the magnitude of PAC between multiple pairs of frequency bands, modulation indices were computed for various frequency bands of 1 Hz width, ranging from and 0.2–5 Hz for the modulating frequency band and 25–60 Hz for the modulated frequency bands (Fig. 1 h). The results were visualized in phase-amplitude comodulograms plots (Fig. 1 h).

To highlight common peaks in coherence between mPFC-Reu and hippocampus-Reu signal pairs, the mean coherence function was computed for two signal pairs: mPFC-Reu and hippocampus-Reu. Mean coherence was obtained by averaging multiple coherence results (n = 39) computed by the mscohere function in MATLAB. To obtain the pseudocolor plot in Fig. 1k, the mean mPFC-Reu coherence was multiplied by coherence values at each frequency of the mean hippocampus-Reu coherence and the resulting matrix was plotted in pseudocolor. To determine possible changes in coherence resulting from the respiration cycle (Fig. 1 m and n), signals were segmented into 2.5 s windows centered to the onset of exhalation. Coherence between two signals was computed via the analytic Morlet wavelet and the mean coherence within 2.5-s windows was calculated for all data.

For time lag analysis, recording samples lasting between 200-500 s were obtained from wake (n = 39 segments), NREM (n = 59 segments) and REM (n = 39 segments) epochs. mPFC, Reu and hippocampal signals were filtered in the gamma range (30–80 Hz) and cross-correlations were computed from the continuous data between mPFC-Reu pairs and hippocampus-Reu pairs of recordings using the xcorr function in MATLAB. Example cross-correlations for mPFC-Reu and hippocampus-Reu pairs in different states of vigilance are shown in Fig. 1j. To measure time lag between two signals, the lag the highest positive peak in the cross-correlogram was measured and compared for wake, NREM and REM recordings (Fig. 1l,m).

### Quantification and statistical analysis

Statistical analyses were performed using the MATLAB Statistics and Machine Learning Toolbox (Mathworks Inc., Natick, MA, USA). One-way ANOVA (MATLAB function *anova1*) was used to compare the means of gamma power and firing rates across states of vigilance and cortical regions. All post-hoc multiple comparisons were done using the Tukey–Kramer method (*multcompare*). Regression was calculated using the least squares method for linear and nonlinear correlations (robust, trust-region-reflective method).

Statistical analysis of the phase of events was performed using the circular statistics tool box developed by Berens^90^. The parameters of interest included the mean resultant vector and the mean resultant vector length (evaluates the circular spread of the data points) for all events. The Rayleigh test was also performed to determine the uniformity in the phase preference of events. Polar plots showing phases, mean phases, and resultant vector lengths of the gamma bursts were plotted using MATLAB.

## Supplementary Information

Supplementary Information.

## Data Availability

Further information and requests for resources and reagents should be directed to and will be fulfilled by the Lead Contact, Igor Timofeev (igor.timofeev@fmed.ulaval.ca). Tetrodes and stainless-steel electrodes manufactured in-house for this study are available from the lead contact with a completed materials transfer agreement. This study did not generate new unique reagents. Original electrophysiological recordings and all code have been deposited at Mendeley Data and are publicly available as of the date of publication. DOIs are listed in the key resources table. Any additional information required to reanalyze the data reported in this paper is available from the lead contact upon request.
