# Supplementary material for: Respiration organizes gamma synchrony in the prefronto-thalamic network
Source: Sci Rep. 2023 May 26;13:8529. doi: 10.1038/s41598-023-35516-7 (PMC10219931; doi:10.1038/s41598-023-35516-7)
Supplement: Supplementary file 1 — Supplementary Information. [file 41598_2023_35516_MOESM1_ESM.docx]

**Respiration organizes gamma synchrony in the prefronto-thalamic network**

Diellor Basha^1,2^, Sylvain Chauvette^2^, Maxim Sheroziya ^1,2^, Igor Timofeev^1,2, *^

^1^Département de psychiatrie et de neurosciences, Université Laval, Québec (Québec) G1V 0A6, Canada

^2^CERVO Centre de recherche, Université Laval, Québec (Québec) G1E 1T2, Canada

*Lead contact and correspondence: igor.timofeev@fmed.ulaval.ca

#
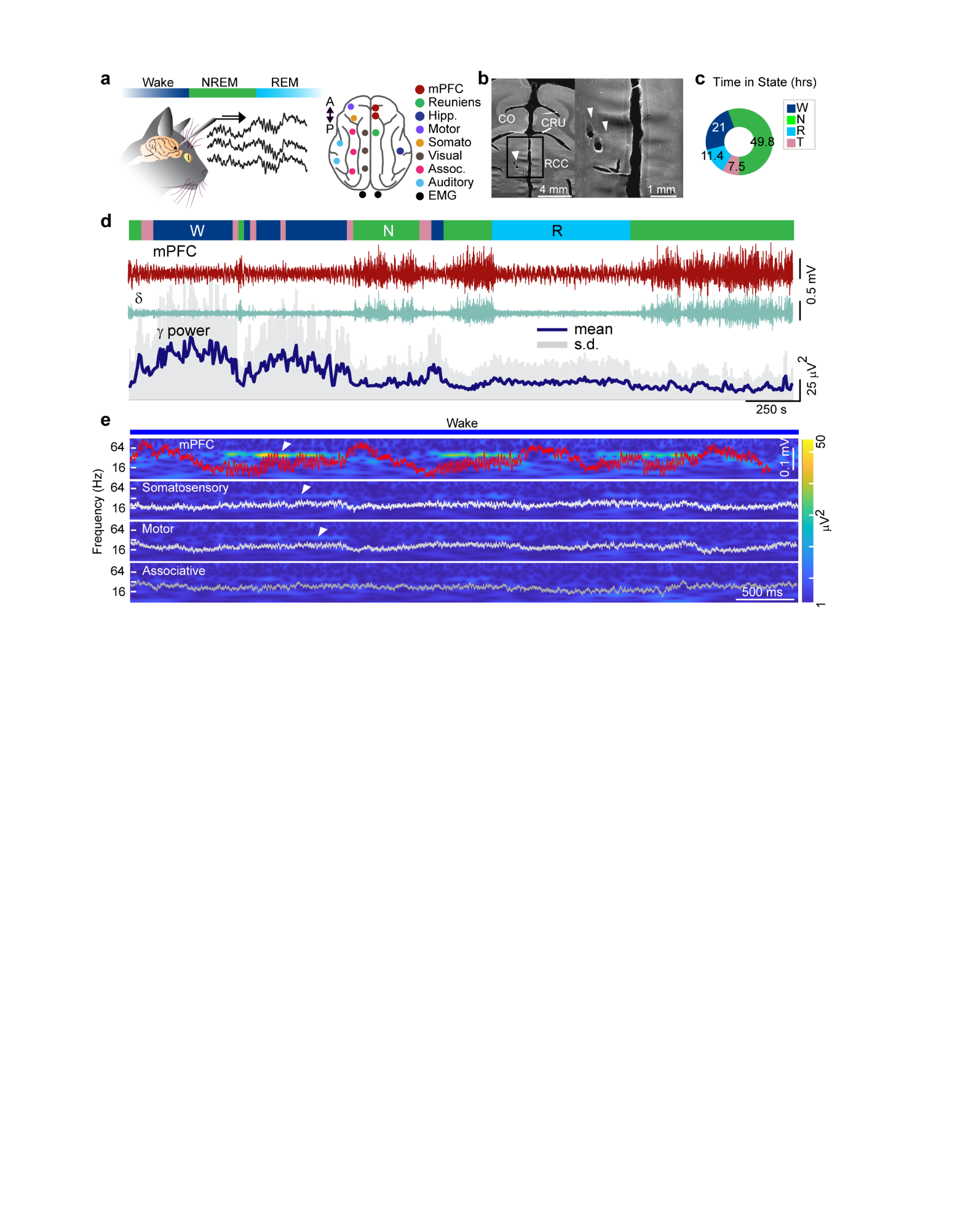
Supplementary- Figures and Legends

Supplementary Fig. S1 Recordings Configuration and prefrontal gamma dynamics

1. An illustration of the recording configuration in head-restrained cats. mPFC recordings were obtained from 4 closely spaced, stainless steel electrodes and contralateral depth recordings of various cortical areas. AP – anterior/posterior
2. A DAPI-labelled, coronal section of the cat frontal lobe showing the dual electrode tracks in deep layers of the mPFC. CO – centrum semiovale, CRU – sulcus cruciatus, RCC- radiation corporis callosi
3. Hours of recordings from each state of vigilance collected from all animals (n= 5 cats). W: Wake, N: NREM sleep, R: REM sleep, T: Transition
4. A sample trace of the raw mPFC local field potential (top, red), the delta bandpassed (1-4 Hz) trace (middle, light green) and the means and standard deviations of 5-second measurements of gamma power of the mPFC signal (bottom, dark blue; gray bars are s.d.). Colored boxes show epochs of wake (blue), NREM (green) and REM (light blue), detected semi-automatically according to mPFC delta, electromyographic and electro-oculographic measurements. Note the decline in gamma power with the emergence of high delta in NREM and the stability of gamma during REM sleep.
5. Sample recording segment and corresponding Morlet wavelet transforms, illustrating high gamma oscillations in the mPFC compared to somatosensory, motor and associative cortical areas.


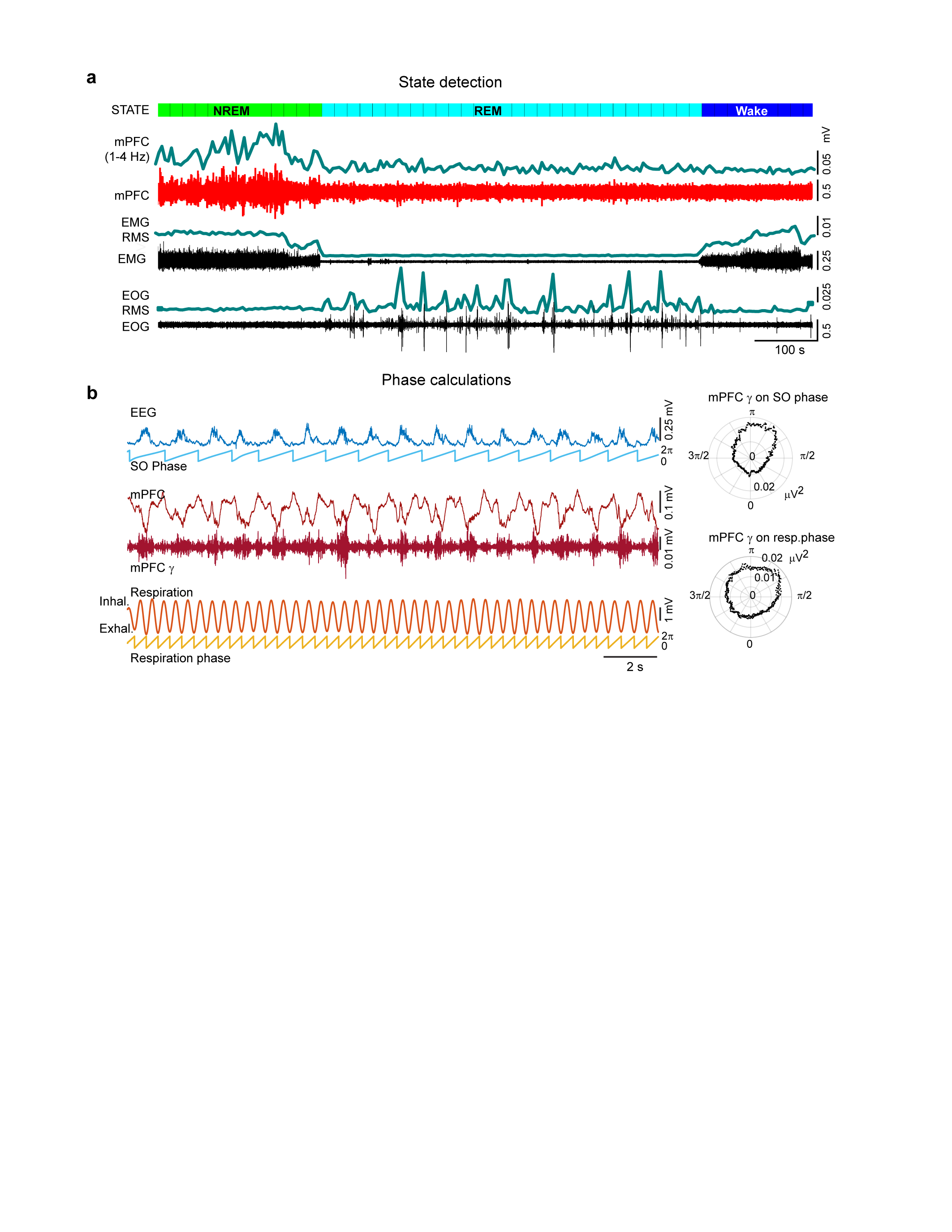
Supplementary Fig. S2 State detection and phase calculations

1. An example recording and the accompanying state detection, showing state transitions through NREM, REM and wake. NREM was detected automatically according to periods of high delta and low muscle power. REM states were scored manually according to low muscle power, low delta and high EOG signal (indicating rapid eye movements) and wake was defined as low delta and high muscle power (see State detection).
2. An example of phase calculation methods in recordings obtained from mice under ketamine-xylazine anesthesia. Signal phase was calculated by taking the angle of the Hilbert-transformed data, bandpassed in the range of interest (0.3-2 Hz for the slow oscillation in anesthesia, 2-5 Hz for respiration in mice, 0.8-2 Hz for respiration in cats).


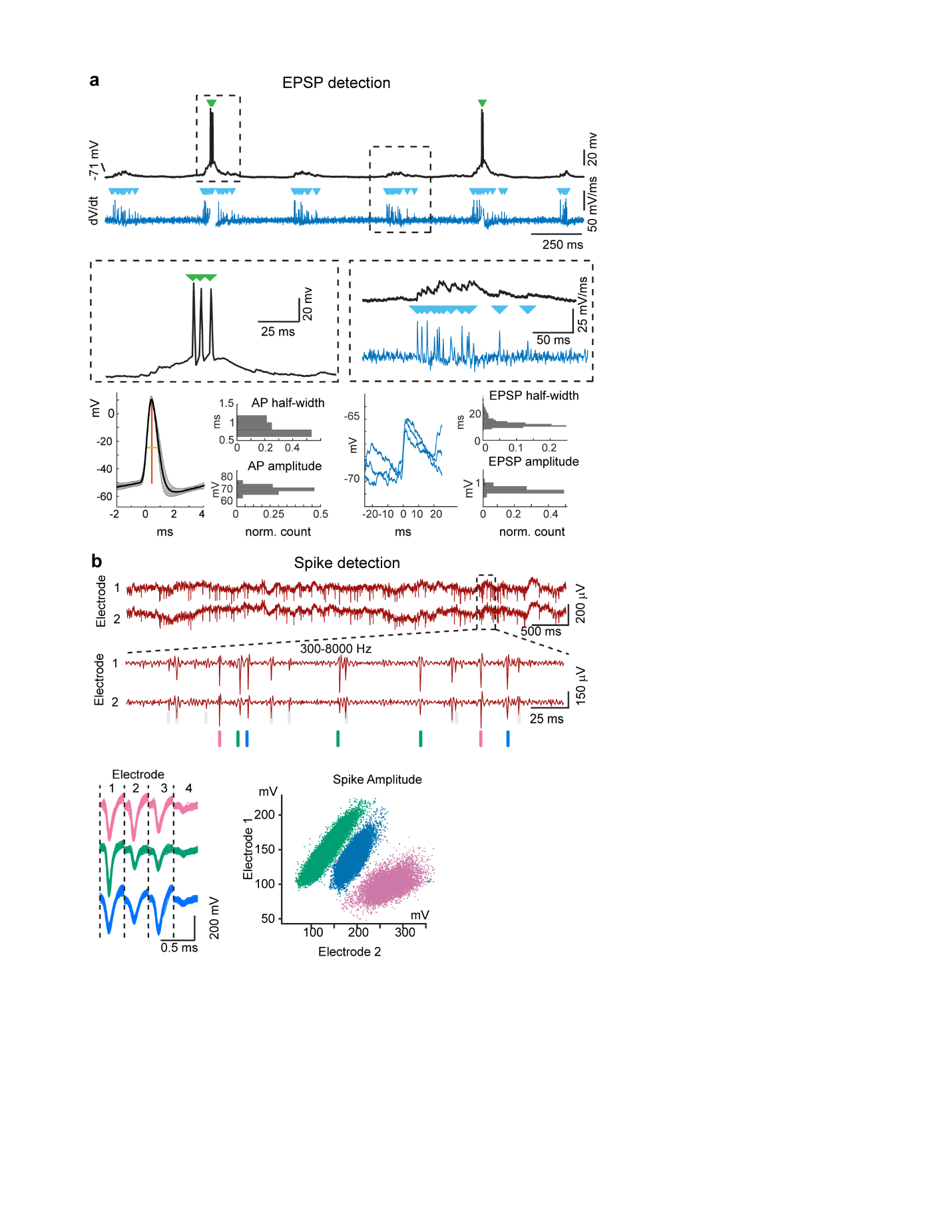
Supplementary Fig. S3 EPSP and spike detection

1. Action potentials were detected as peaks in the Vm signal 10 s.d. above the mean (green triangles). To detect EPSPs, the Vm was lowpassed (<1000 Hz) and differentiated to obtain dV/dt values. Peaks above mean + 1 to 3 s.d. of the differentiated signal were detected as EPSPs (blue triangles). Example distribution of action potential half-width duration, amplitude and EPSP half-width duration, amplitude.
2. Single-units were detected using template matching protocols in Spike2 and discriminated according either to principal component analysis or amplitude-amplitude clustering from recording pairs of the same tetrode.


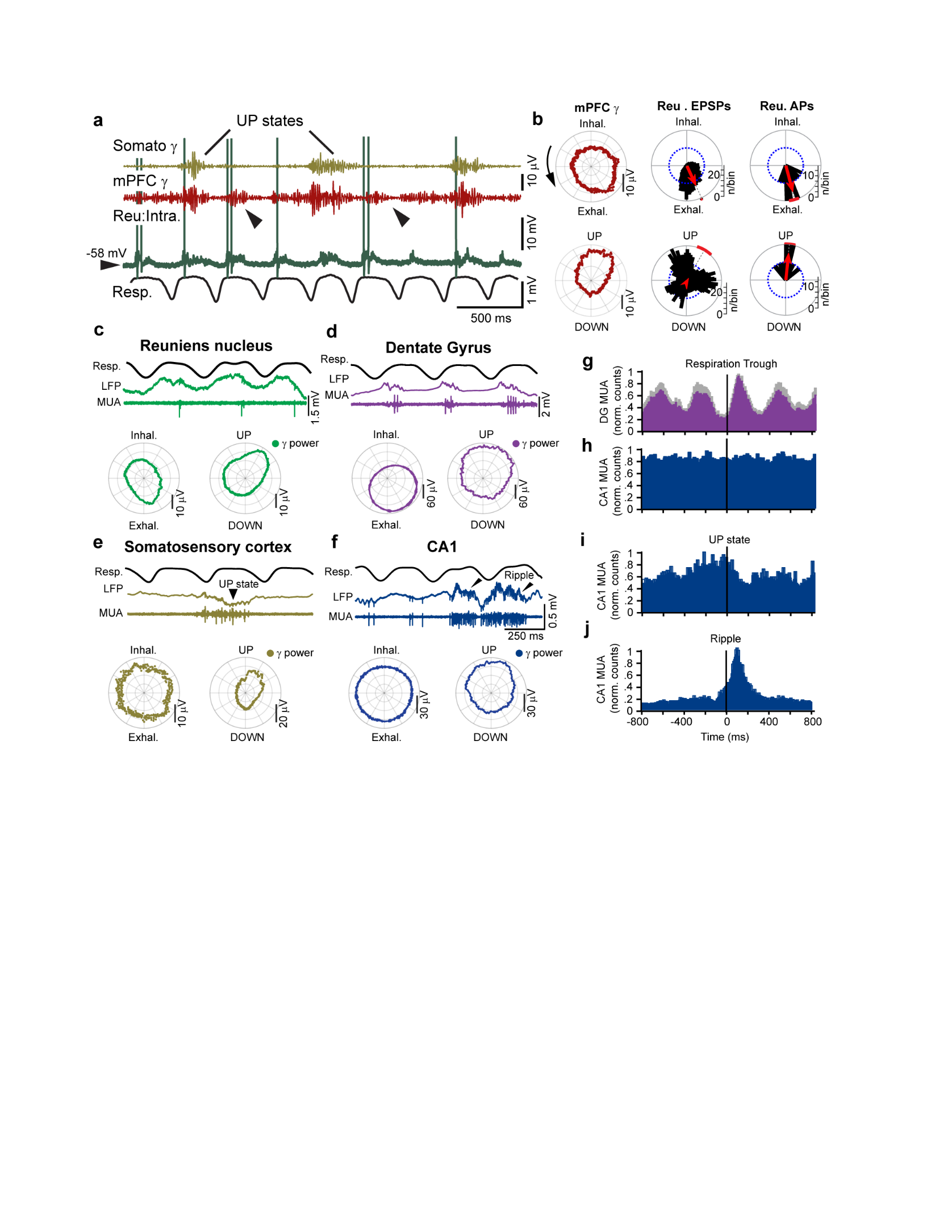
Supplementary Fig. S4 Spike and gamma oscillatory dynamics in the mPFC, reuniens and the hippocampus

1. An example intracellular recording of the reuniens nucleus and bandpassed (30-80 Hz) LFP recordings of the mPFC and somatosensory cortex. The main determinant of gamma modulation in the somatosensory cortex was the slow oscillation. In the mPFC, gamma amplitude was modulated by the slow oscillation but also increased transiently with respiration, following action potential discharges in Reu.
2. Polar plots showing the modulation of mean gamma amplitude by the slow oscillation and weak modulation by respiration. EPSPs and action potentials in Reu were strongly modulated by respiration and preceded the gamma maxima in the mPFC.
3. An example LFP trace of Reu and its bandpassed (300-8000 Hz) derivative, showing the modulation of multiunit activity in the reuniens by respiration. Gamma amplitude was modulated by respiration and the slow oscillation.
4. An example LPF trace of the dentate gyrus and the corresponding multiunit activity, phase-locked to respiration. Gamma power in the dentate gyrus was strongly modulated by respiration, reaching its maximum in the transition from exhalation to inhalation.
5. An example LFP trace of the somatosensory cortex and the corresponding multiunit activity, showing a lack of firing modulation by respiration. The main determinant of firing rate and gamma amplitude modulation in the somatosensory cortex was the slow oscillation.
6. An example LFP trace of CA1 region of the hippocampus showing that the main contributor to firing rate modulation is the sharp-wave ripple. Gamma power in the mPFC was not modulated by respiration and weakly modulated by the slow oscillation.
7. Peri-event time histogram showing multi-unit activity from the dentate gyrus, referenced to the trough of respiration signal (exhalation). Multi-unit activity in the dentate gyrus was strongly modulated by respiratory cycles.
8. Peri-event time histogram of multi-unit activity of the CA1, referenced to exhalation peak in the respiratory signal. CA1 multi-unit activity showed no phase-locking to respiration.
9. Peri-event time histogram of multi-unit activity of the CA1, referenced to the onset of UP states detected in from the contralateral EEG. CA1 multi-unit activity decreased at the onset of cortical UP states.
10. Peri-event time histogram of the multi-unit activity from the CA1, referenced to the onset of hippocampal sharp-wave ripples recorded from the same electrode. CA1 multi-unit activity increased strongly at the onset of hippocampal SWRs.


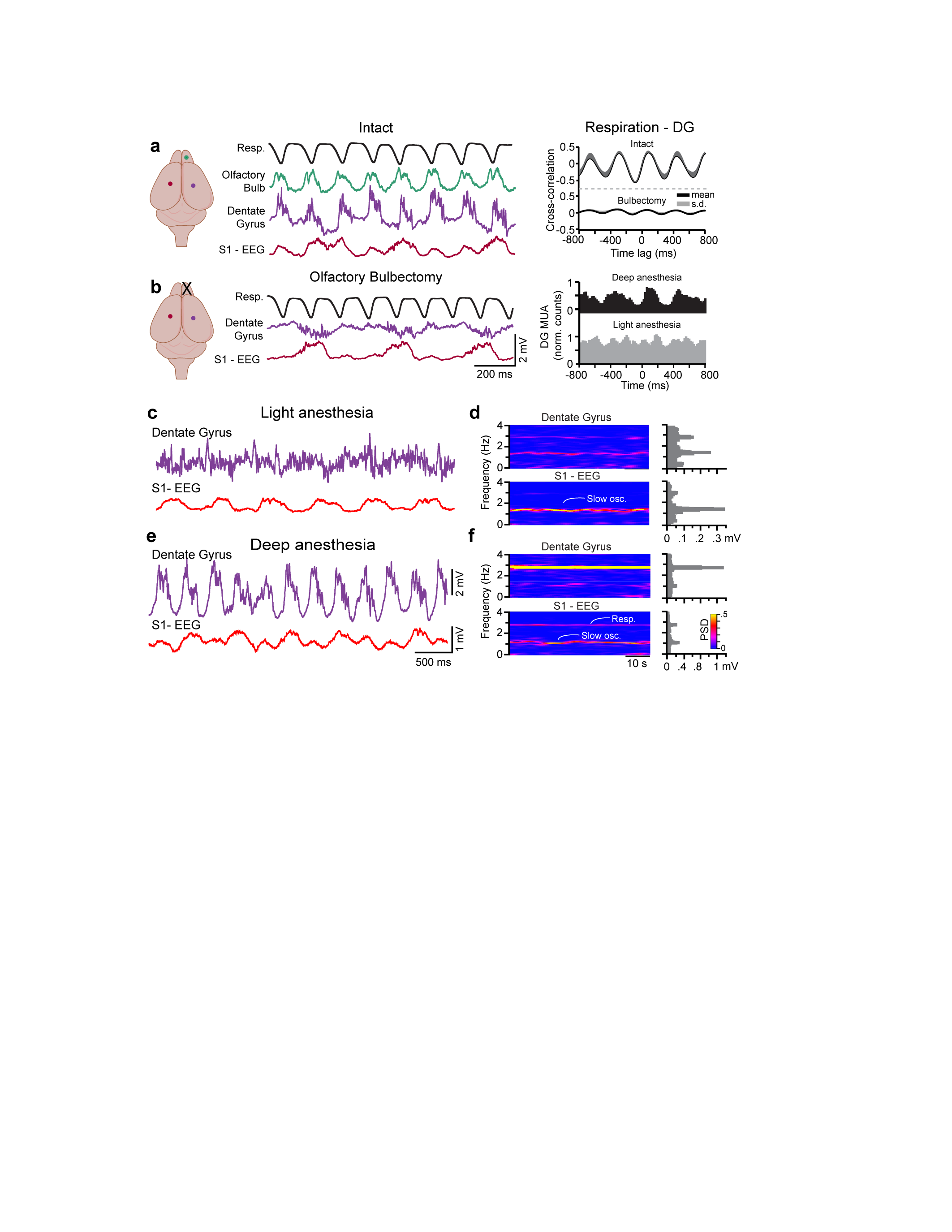
Supplementary Fig. S5 Respiratory rhythms in the dentate gyrus depend on olfactory input and levels of anesthesia

1. A sample LFP recording of the dentate gyrus and the olfactory bulb showing rhythmic modulation of amplitude with respiration.
2. A sample LFP recording of the dentate gyrus following the ablation of the ipsilateral olfactory bulb. Respiratory modulation of the dentate gyrus diminished following bulbectomy.
3. A sample LFP recording of the dentate gyrus under light ketamine-xylazine anesthesia showing weak modulation of amplitude with respiration.
4. Spectrogram of the dentate gyrus LFP and S1-EEG showing that the primary contributor of spectral power under light anesthesia is the delta oscillation.
5. Sample LFP recording of the dentate gyrus under deep ketamine-xylazine anesthesia. The main frequency of the dentate gyrus LFP is the 3-4 Hz respiratory cycle (F).
